# Characteristics of salivary gland tumours in the United Arab Emirates

**DOI:** 10.3332/ecancer.2015.583

**Published:** 2015-10-20

**Authors:** Yasir Al Sarraj, Satish Chandrasekhar Nair, Ammar Al Siraj, Maher AlShayeb

**Affiliations:** 1, 4 Ajman University of Science and Technology, Department of Oral and Maxillofacial Surgery, Post Box 346, United Arab Emirates; 2 Tawam Hospital- Johns Hopkins Medicine International Affiliate, Department of Academic Affairs-Medical Research, Post Box 15258, Al Ain, United Arab Emirates; 3 Mawi Medical Centre, Post Box 55510, Dubai, United Arab Emirates

**Keywords:** salivary gland tumour, neoplasm, Gulf, UAE

## Abstract

Salivary gland tumours (SGT) are relatively rare cancers characterised by striking morphological diversity and wide variation in the global distribution of SGT incidence. Given the proximity to the head and neck structures, management of SGT has been clinically difficult. To the best of our knowledge, there are no epidemiological studies on SGT from the United Arab Emirates (UAE) or the Gulf Cooperation Council Countries (GCC). Patient charts (N = 314) and associated pathological records were systematically reviewed between the years 1998–2014. Predominance of benign (74%) compared with malignant (26%) SGT was observed. Among the 83 malignant SGT identified, frequency was higher in males (61%) than in females (39%) and peak occurrence was in the fifth decade of life. Mucoepidermoid carcinoma was the most common type of tumour (35%) followed by adenoid cystic carcinoma (18.1%) and acinar cell carcinoma (10.8%). A similar pattern of tumour distribution was seen in patients from GCC, Asian, and Middle East countries. This is the first report to address the distribution of salivary gland tumours in a multiethnic, multicultural population of the Gulf. The results suggest that the development of an SGT registry will help clinicians and researchers to better understand, manage, and treat this rare disease.

## Introduction

Salivary gland tissues are distributed in the upper aerodigestive tract; the parotid, submandibular, and sublingual are the major salivary glands [1]. The minor salivary glands are present in other sites such as lips, palate, tongue, oropharynx, etc. [1]. Salivary glands contain many different types of cells that can become malignant leading to many different types of salivary gland cancer. Globally, salivary gland tumours are relatively rare cancer characterised by a striking range of morphological diversity within and between different tumour types [1]. These rare heterogeneous lesions with complex clinical and pathologic characteristics and dissimilar biological behaviour account for 0.4–13.5 cases per 100,000 people [2]. Given the proximity to the various important head and neck structures, clinical management of SGT is also challenging [2]. The diversity of SGT lead to disagreements over histological nomenclature which were resolved in 1971 by the World Health Organisation (WHO) histological classification system, revised in 1999 and 2005 [3].

The Gulf Cooperation Council (GCC) member countries of Oman, Bahrain, Kuwait, Qatar, Saudi Arabia, and the United Arab Emirates experienced a significant increase in wealth from natural resources, including oil and natural gas in the past forty years [4]. This rise in prosperity led to population growth, swift urbanisation, and subsequent changes in lifestyle. Lifestyle-related diseases, namely diabetes, cardiovascular disease, and cancer soon followed [5]. The UAE is a multiethnic, multi-cultural nation with a population of almost eight million at a ratio of 1:7 (UAE nationals to expatriates) [6]. Abu Dhabi, the capital and the largest among the seven emirates of the UAE, and Dubai have witnessed a surge in the number of expatriates from all over the world, especially Asia, Middle East, Africa, and Europe since the year 2000 [7]. With the rapidly increasing population, the region has seen an increase in the number of cancer deaths, especially in the fifth and sixth decades of life, both for expatriates and nationals [8].

Striking epidemiological differences in the global distribution of SGT have been observed [1]. The etiology of SGT are not clearly known although genetic predisposition, viral infections, cigarette smoking, and exposure to environmental chemical hazards have been postulated [2]. Although, SGT predominantly arise in patients closer to 45 years of age, the sex ratio varies according to the tumour type. A wide range of variation follows site distribution, incidence, and histological types of SGT, although pleomorphic adenoma is reported to be the most common [2]. Adenoid cystic carcinoma and mucoepidermoid carcinoma are the most common malignant SGT [2, 7]. The parotid gland, followed by the submandibular gland, and the palate are most often affected by the SGT and the sublingual being the least [2].

## Materials and methods

A retrospective review of medical records was conducted and corresponding pathological data from the Comprehensive Cancer Registry (CCR) for a period of over 25 years from 1998 until 2014 [9]. The CCR is located at Tawam Hospital, a 468-bed tertiary care-teaching hospital, Joint Commission accredited, and a regional oncology referral centre until two years ago, still a referral centre for the emirates of Abu Dhabi. Oncology patients from within the UAE, the Gulf countries, and from Asia and Africa are referred to the hospital, and the CCR has more than 21,000 records of cancer cases until to date [9]. An oncology service is provided to UAE nationals and non-nationals possessing a National Insurance Card since 2012, although exemptions are made for rare-diseases and sub-specialty treatment such as radiation oncology. The present study was conducted following approval from the local central ethics committee (AAMDHREC#12/13).

Eighty-three patients with primary neoplasm of the salivary gland were identified which were further analysed at the Maxillofacial Department and the Pathology Department in Ajman University of Science and Technology. The clinical and histopathological records were reviewed by the authors in consultation with a pathologist and a surgeon. The major salivary glands involved the parotid gland, submandibular gland, and sublingual gland whereas the minor glands included those in the lip and intra-oral regions (the palate, lip, buccal cavity, floor of the mouth, and the maxillary region). The data were evaluated for all patients with surgically removed or biopsied pathologies of the salivary gland. Corresponding data was collected regarding histological diagnosis, age, gender, nationality of patients, and site and metastasis of neoplasm from which the biopsy was taken. The histological specimens were reviewed by the pathologist, staged, and ranked accordingly [10]. Inter-rater reliability between the pathologists was assessed using Cohen’s kappa and was >0.80 [11]. Data was statistically analysed using the SPSS programme (IBM SPSS Statistic 20).

## Results

Review of medical and pathology records over a twenty-five year period collected 314 cases of salivary gland tumours. Among the salivary gland tumours, predominance was for benign (74%) type (Table 1). Pleomorphic adenoma (64%) and Warthin’s tumour (24.9%) constituted majority of the benign major salivary gland tumour. Twenty-six percent (n = 83) of the total SGT presented with malignant primary salivary gland neoplasms (Table 1), occurring unilaterally. The site of distribution data showed that the majority of the malignant SGT were in the parotid glands (n = 63), followed by the submandibular glands (n = 15), and (n = 1) in the sublingual gland (Table 1). Minor salivary gland tumours were not detected in the present study. Among the 83 cases identified, malignant SGT was observed more frequently in males 61% (n = 51) than in females 39% (n = 32). The peak occurrence of SGT was in the fifth decade of life both for males and females. The average age (± standard deviation) was 42.8 ± 5 and 41.9 ± 19, not statistically different for SGT between males and females, and ranged widely from 3–79 years.

**Figure 1.**
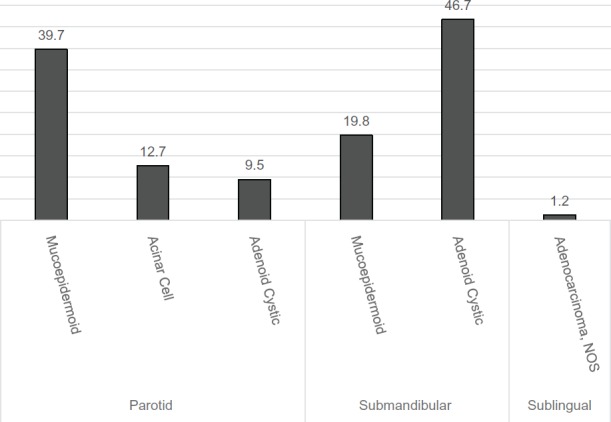
Malignant types and distribution of the various tumours in the major salivary gland. Values expressed in percentages.

Histopathological distribution of the malignant salivary tumour indicated mucoepidermoid carcinoma was the most common type of tumour and constituted approximately (35%) of all tumours. This was followed by adenoid cystic carcinoma (18.1%) and acinar cell carcinoma (10.8%) (Table 2). Each of the other type of tumours such as basal cell adenocarcinoma, follicular lymphoma, squamous cell carcinoma, duct carcinoma, etc. contributed to less than 5% to the total number of malignant SGT. Among the parotid gland tumours, the most frequent malignant tumour was mucoepidermoid carcinoma (39.7%) followed by acinar cell carcinoma (12.7%) and adenoid cell carcinoma (9.5%). In stark contrast, the submandibular gland tumours were largely adenoid cystic carcinomas (46.7%) and mucoepidermoid carcinomas (19.8%). In the sublingual gland, the only malignant tumour observed was adenocarcinoma, not otherwise specified (NOS) (Figure 1). Primary sites could not be determined for four of the cases.

Per American Joint Committee on Cancer (AJCC) staging criteria, most cancers were detected at stage 2 (28.9%, n = 24), followed by stage 3 (19.2%, n = 16), stage 1 (9.6%, n = 8), and stage 4 (4.8%, n = 4). Thirty-one tumours (37%) were not staged because of incomplete information in the patient medical records or pathological information (data not shown).

**Figure 2.**
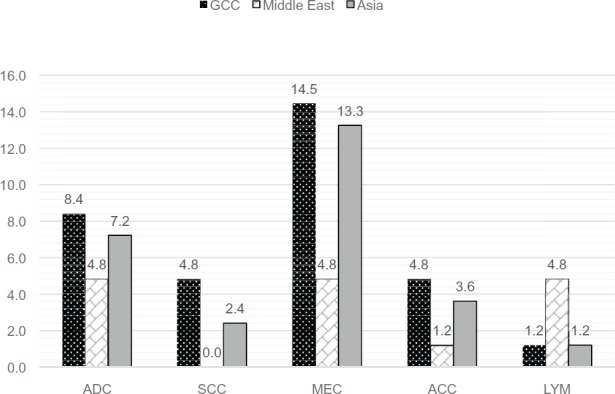
Distribution of malignant salivary gland tumours among the multi-ethnic population of the UAE. Values expressed in %. ADC = Adenocarcinoma, SCC – Squamous Cell Carcinoma, MEC = Mucoepidermoid Carcinoma, ACC = Acinar Cell Carcinoma and LYM = Lymphoma.

Mucoepidermoid carcinoma (14.5%, n = 12) was more common in patients from the Gulf Cooperation Countries and from Asia (13.2%, n = 11). Similar pattern of distribution was seen both for adenocarcinoma and acinar cell carcinoma (Figure 2). Lymphoma was high among the Middle Eastern population (excluding the GCC).

## Discussion

The present study reviewed 314 cases of salivary gland tumours in the past twenty-five years (1998–2014) using medical records, both in electronic and paper formats. A Hospital Information System (or Malaffi which means ‘my_file’ in Arabic) was implemented in 2008 [7]. Among the salivary gland tumours, predominance was for benign (74%) type. Pleomorphic adenoma (64%) constituted the majority of the benign major salivary gland tumours, followed by Warthin’s tumour (24.9%), consistent with the WHO report [12], although this is lower than in Denmark [13]. Of note a predominance of benign SGT in the range between 60–80% have been reported from different parts of the world [14–15].

The frequency for malignant SGT in our study was 26%, lower than reported from other Middle Eastern and Asian countries such as Iran (32%) and Jordan (32%), Sri Lanka (50%), India (38%), China (32%) in Asia, Nigeria (43%) and Uganda (46%) in Africa [15–16]. In our study, there was a higher proportion of malignant SGT in males (61%) than in females (39%). The age of the patients in this study ranged from 3 to 79 years, with the mean and standard deviation equal to 42.4 ± 6.6 years (male and female combined). The peak occurrence for malignant salivary gland tumours was the fifth decade of life, both for males and females. The gender ratio and age range resembled observations from a study involving a closely related Middle Eastern population [16–17].

Our study showed that mucoepidermoid carcinoma was the most common type of tumour and constituted approximately (35%) of all salivary gland tumours, followed by adenoid cystic carcinoma and acinar cell carcinoma. Mucoepidermoid carcinoma was significantly higher in Asian countries such as Sri Lanka (22%) and India (25%) [17]. The frequency of mucoepidermoid carcinoma was lower in the Middle East [Iran (11%) and Jordan (7%)] compared to Congo (8%) and Uganda (9%) in Africa [16–17]. Interestingly, the pattern of distribution of mucoepidermoid carcinoma, adenoma, and acinar cell carcinoma were comparable between the population of the Gulf and Asia, in spite of geographic isolation of the subpopulations from the Gulf and the practice of consanguineous marriages [18]. Stage 2 was the most frequent malignant salivary gland tumour observed in our study. Predominance of stage 2 and stage 3 were seen among GCC (45.8% and 56.2%) and Asian (29.1 and 25.0%) nationalities. Prognosis studies indicate a 60–70% survival rate post diagnosis with stage 2 and stage 3 malignant salivary gland tumours [19–20]. Both GCC and Asians showed 50% frequency each for Stage 4 which has the poorest prognosis outlook and less than 30% survival beyond ten years post diagnosis of the tumours [19–20].

Our study has several limitations, foremost is the fact that we could identify only 83 malignant salivary gland tumours for a span of over 25 years. Part of this limitation could result from under reporting and issues with the differential diagnosis given the rarity of the SGT, especially in the earlier years before electronic hospital information systems. There may also be a possibility of inaccurate pathological staging of the disease prior to the recent revision by the WHO in 2005, the period where incomplete staging information was found in the 31 patients and pathological records then had to be excluded.

## Conclusion

In summary, salivary gland tumours comprise a highly heterogeneous histopathologic group of tumours and given their proximity to the head and neck structures, this makes clinical management of these cases difficult. There have been no epidemiological studies for benign and malignant salivary gland tumours in the UAE or the Gulf, regions of high population diversity. This is the first report to address the distribution of salivary gland tumours in the UAE. The results suggest that a salivary gland tumour registry will help to better understand, document, manage, and treat this rare disease, globally and in the Arabian Gulf region.

## Author contribution

Both YAS and SCN contributed equally to this study.

## Conflicts of interest

The authors report no conflict of interest.

## Funding support

This study was not supported by funding from any sources.

## Figures and Tables

**Table 1.** Assessment of malignant salivary gland tumours in the UAE.

Total salivary gland tumours (N = 314)	n (%)
Benign salivary gland tumours	231 (74)
Malignant salivary gland tumours	83 (26)
Malignant SGT	
Parotid	63 (76)
Submandibular gland	15 (18)
Sublingual gland	1 (1)
Minor salivary gland	0 (0)
Male	51 (61)
Female	32 (39)

**Table 2.** Histopathological distribution of salivary gland tumours (n = 83).

Salivary tumour types	Frequency	Percentage
Mucoepidermoid carcinoma	29	34.9
Adenoid cystic carcinoma	15	18.1
Acinar cell carcinoma	9	10.8
Lymphoma, malignant, large B-cell	4	4.8
Squamous cell carcinoma, keratinising, ^*^NOS	3	3.6
Adenocarcinoma, ^*^NOS	2	2.4
Squamous cell carcinoma, ^*^NOS	2	2.4
Carcinoma in pleomorphic adenoma	2	2.4
Duct carcinoma, ^*^NOS	2	2.4
Mixed tumour, malignant, ^*^NOS	2	2.4
Epithelial–myoepithelial carcinoma	2	2.4
Basal cell adenocarcinoma	1	1.2
Lymphoma, follicular, ^*^NOS	1	1.2
Eccrine adenocarcinoma	1	1.2
Squamous cell carcinoma, lg cell, keratinising	1	1.2
Lymphoma, follicular, grade 2	1	1.2
Polymorphous low grade adenocarcinoma	1	1.2
Synovial sarcoma, biphasic	1	1.2
Clear cell adenocarcinoma, ^*^NOS	1	1.2
Lymphoepithelial carcinoma	1	1.2
Carcinoma, ^*^NOS	1	1.2
Carcinoma, undifferentiated, ^*^NOS	1	1.2

* NOS (Not otherwise specified)
